# Deformation Behavior and Precipitation Features in a Stretched Al–Cu Alloy at Intermediate Temperatures

**DOI:** 10.3390/ma13112495

**Published:** 2020-05-29

**Authors:** Y.C. Lin, Wen-Yong Dong, Xu-Hao Zhu, Qiao Wu, Ying-Jie He

**Affiliations:** 1 School of Mechanical and Electrical Engineering, Central South University, Changsha 410083, China; mschen77@163.com (W.-Y.D.); ychuang63@163.com (X.-H.Z.); Xianyangwu88@163.com (Q.W.); lingli855@163.com (Y.-J.H.); 2 State Key Laboratory of High Performance Complex Manufacturing, BP156, Changsha 410083, China

**Keywords:** Al alloy, deformation behavior, precipitation features, phases

## Abstract

Deformation behavior and precipitation features of an Al–Cu alloy are investigated using uniaxial tensile tests at intermediate temperatures. It is found that the true stress drops with the decreased strain rate or the increased deformation temperature. The number of substructures and the degree of grain elongation decrease with the raised temperature or the decreased strain rate. At high temperatures or low strain rates, some dynamic recrystallized grains can be found. The type of precipitates is influenced by the heating process before hot tensile deformation. The content and size of precipitates increase during tensile deformation at intermediate temperatures. As the temperature increases over 200 °C, the precipitation process (Guinier Preston zone (G.P. zones)→θ′′ phase→θ′ phase) is enhanced, resulting in increased contents of θ′′ and θ′ phases. However, θ′′ and θ′ phases prefer to precipitate along the {020}_Al_ direction, resulting in an uneven distribution of phases. Considering the flow softening degree and the excessive heterogeneous precipitation of θ′′ and θ′ phases during hot deformation, the reasonable strain rate and temperature are about 0.0003 s^−1^ and 150 °C, respectively.

## 1. Introduction

In view of their excellent properties, 2xxx series Al (Al–Cu) alloys are widely used for aerospace components [1,2,3,4,5]. For instance, rocket engine fuel tanks are usually manufactured using 2xxx Al alloys [6]. Generally, the tanks are of large diameter and small thickness, and they are often formed by tensile deformation at room temperature. However, during tensile deformation, the work hardening is usually very obvious, and uneven microstructures are induced. Therefore, it is essential to find reasonable deformation parameters (deformation temperature and strain rate) to relieve work hardening and optimize microstructures.

In recent years, many researches on the deformation behavior [7,8,9,10,11] and precipitation features [12,13,14,15] of Al–Cu alloys were carried out. Liu et al. [16] described the continuous dynamic recrystallization behavior of a compressed Al–Cu alloy at the temperature range of 350–500 °C. Chen et al. [17] researched the high-temperature compressive features of an Al–Cu alloy. Liu et al. [18] found that the main softening mechanism of a compressed 2219 aluminum alloy is dynamic recovery at the temperature range of 250–500 °C. Wei et al. [19] analyzed the morphological changes of S-phase in a high-temperature crept 2024 aluminum alloy under tensile stress. Lin et al. [6] discussed the effects of cooling rate and solution time on the precipitated transformation of an Al–Cu alloy, and they found that the precipitation of θ′ and θ′′ phases is enhanced with increased cooling rate and solution time. García-Hernández et al. [20] discovered that fine homogeneous precipitates in a 2024 Al alloy with a thickness of 8 mm can be acquired when the thickness reduction is 15%. Song et al. [21] found that the dimensional stability of an Al–Cu–Mg alloy can be improved by stress-aging. Paoletti et al. [22] and Li et al. [23] modeled the creep behavior of an aged Al–Cu–Mg alloy. Li et al. [24] discussed the synergy influence of pre-straining and Si addition on microstructures of Al–Cu–Mg alloys. Mirzadeh [25] studied the complex hot deformation mechanisms of 7075 and 2024 Al alloys, as well as developed accurate physically based constitutive models to depict their flow behavior. 

The above researches mostly concentrated on the deformation behavior and precipitation features in hot compressed Al–Cu alloys, where the deformation temperature is generally higher than 250 °C. However, few investigations focused on the tensile deformation at intermediate temperatures such as below 250 °C. Nevertheless, the researches on the deformation behavior and precipitation features in Al–Cu alloys such as 2219 aluminum alloy at intermediate temperatures are significant for the industrial production of large-scale thin-walled ellipsoidal heads. In this work, the deformation behavior of an Al–Cu alloy is investigated by uniaxial tensile tests at intermediate temperatures (100–250 °C). The changes in substructures and grains were analyzed by electron backscattered diffraction (EBSD) analysis. Furthermore, the precipitation features during tensile deformation were investigated by differential scanning calorimetry (DSC), transmission electron microscopy (TEM), and scanning electron microscopy (SEM).

## 2. Materials and Experimental Procedures

**Figure 1:**
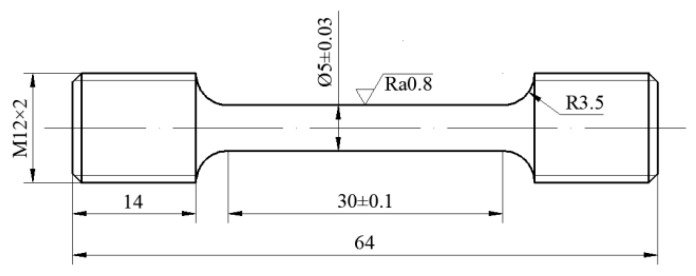
Specific dimensions of cylindrical tensile specimens (unit: mm).

The initial experimental material was one typical Al–Cu alloy (2219 aluminum alloy) rolled sheet with 12 mm thickness. Its chemical composition was 6.37 Cu–0.30 Mn–0.16 Fe–0.12 Zr–0.05 Si–0.04 Ti–Al (balance) (wt. %). The sheet was solution-treated at 535 °C for 40 min [6]. Figure 1 displays the cylindrical specimens cut from the sheet along the rolling direction. The tensile deformation experiments were executed on an MTS-GWT2105 test machine (MTS company; Shanghai, China). The experimental temperatures were 100, 150, 200, and 250 °C. The strain rates were 0.00003, 0.0003, and 0.003 s^−1^. The samples were firstly heated to experimental temperatures by 10 °C/min and then held for 5 min to acquire an even temperature field. Afterward, the samples were stretched to 15% true strain at different strain rates. Finally, the samples were cooled in air to room temperature.

To analyze the effects of deformation parameters on microstructures, foils were cut from the central part of stretched samples. The foils were polished by abrasive paper and then thinned by electro-polishing in a solution of HNO_3_ (240 mL) and CH_3_OH (560 mL) at −30 °C. The prepared foils were analyzed by EBSD (Helios Nanolab 600i; FEI company; Hillsboro, OR, USA) and TEM (Tecnai G2 F20; FEI company; Hillsboro, OR, USA) detectors. Additionally, the content and type of precipitated phases were analyzed by DSC (DSC8500; PerkinElmer company; Waltham, MA, USA). Firstly, to acquire the baseline of DSC, an empty crucible was heated with the heating rate of 10 °C/min. Then, the samples were grinded with sandpaper and cleaned by anhydrous ethanol. The size of DSC samples was 5 mm × 5 mm × 2 mm, and the mass was between 90 and 110 mg. Next, the samples were put in a crucible, and these samples were subsequently heated with the same heating rate. To evaluate the mechanical properties of the alloy, the uniaxial tensile tests were executed on the MTS-GWT2105 test machine in the tested condition. After tensile deformation, the fracture morphologies were observed using a JEOL-7001F1 field emission (FE) SEM (FEI Electron Optics B.V; Prague, Czech Republic).

## 3. Experimental Findings and Discussions

### 3.1. Deformation Behavior and Mechanisms

**Figure 2:**
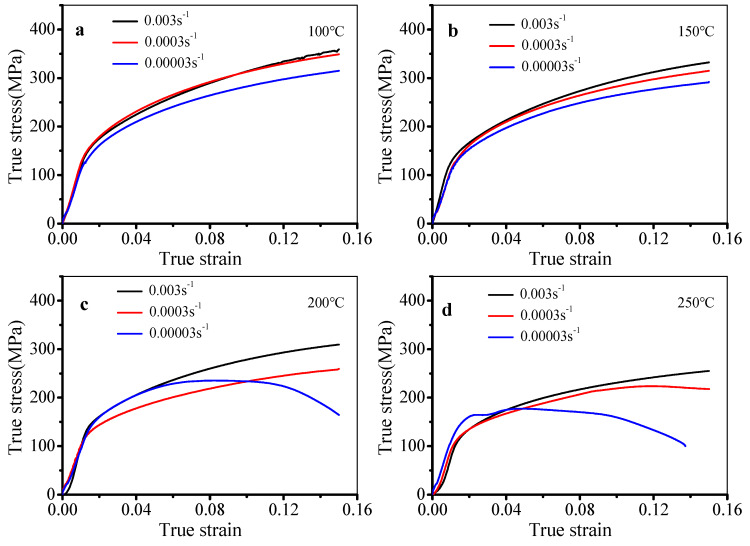
Flow stress curves at (**a**) 100 °C, (**b**) 150 °C, (**c**) 200 °C, and (**d**) 250 °C.

The flow stress curves of the stretched alloy are displayed in Figure 2. The stretched process can be divided into the elastic and plastic deformation stages. In the elastic deformation stage, the true stress linearly rises with the true strain. This is because dislocations quickly accumulate in material. Meanwhile, the rearrangement and annihilation of dislocations are low because of the short deformation time [26,27]. However, in the plastic deformation stage, the slope of the flow stress curve gradually decreases with increased strain. The reason is that the work hardening (WH) gradually weakens, while the dynamic softening is gradually enhanced [28,29]. In Figure 2a,b, the true stress gradually rises with the increased true strain at 100 °C and 150 °C. This is because WH is dominant, but the role of dynamic softening is relatively low at low temperatures [30,31]. In Figure 2c, as the temperature is raised to 200 °C, the true stress still rises with the raised true strain at 0.003 s^−1^ and 0.0003 s^−1^. However, it firstly increases and then keeps stable, finally rapidly dropping with the increase in strain at 0.00003 s^−1^, i.e., the dynamic softening is enhanced. In Figure 2d, as the temperature is further increased to 250 °C, the true stress increases with the increase in strain at 0.003 s^−1^. The true stress firstly rises and then keeps stable at 0.0003 s^−1^. However, obvious dynamic softening behavior is observed at 0.00003 s^−1^. In general, the true stress is affected by strain rate and temperature. The true stress declines with the increased temperature. The reason is that the thermal motion of atoms is enhanced, which decreases the bonding force of atoms, and plastic deformation easily occurs [25,32,33]. Thus, the role of dynamic softening is more obvious at higher temperatures. In addition, the true stress drops with the decreased strain rate, because the deformation time is enough at low strain rates, enhancing the annihilation and rearrangement of dislocations [34,35]. Then, the true stress drops with decreasing strain rate.

**Figure 3:**
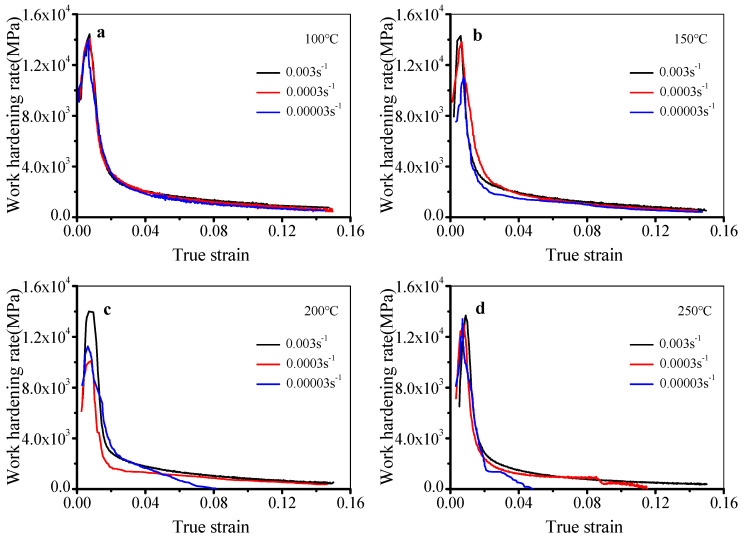
Work hardening rate–true strain curves at (**a**) 100 °C, (**b**) 150 °C, (**c**) 200 °C, and (**d**) 250 °C.

Usually, the work hardening rate is used to depict work hardening behavior in deformed alloys. The plots of work hardening rate and true strain in diverse deformation conditions were acquired by differentiating the flow stress curves, as presented in Figure 3. In the elastic deformation stage, the work hardening rate quickly rises. Then, the work hardening rate drops with increasing strain in the plastic deformation stage. In Figure 3a–d, it can be noted that the maximum work hardening rate is smaller at lower strain rates or higher temperatures. Meanwhile, the work hardening rate quickly drops in the plastic deformation stage. In most deformation conditions (Figure 3a–c), the work hardening rate does not decrease to zero with the raised strain, except at 200 °C and 0.00003 s^−1^. In Figure 3d, the temperature is raised to 250 °C, while the work hardening rate decreases to zero at 0.0003 s^−1^ and 0.00003 s^−1^ when the true strains are 0.115 and 0.048, respectively. This reveals that the influence of dynamic softening is strong at lower strain rates or higher temperatures. 

### 3.2. Effects of Deformation Parameters on Substructures and Grains

**Figure 4:**
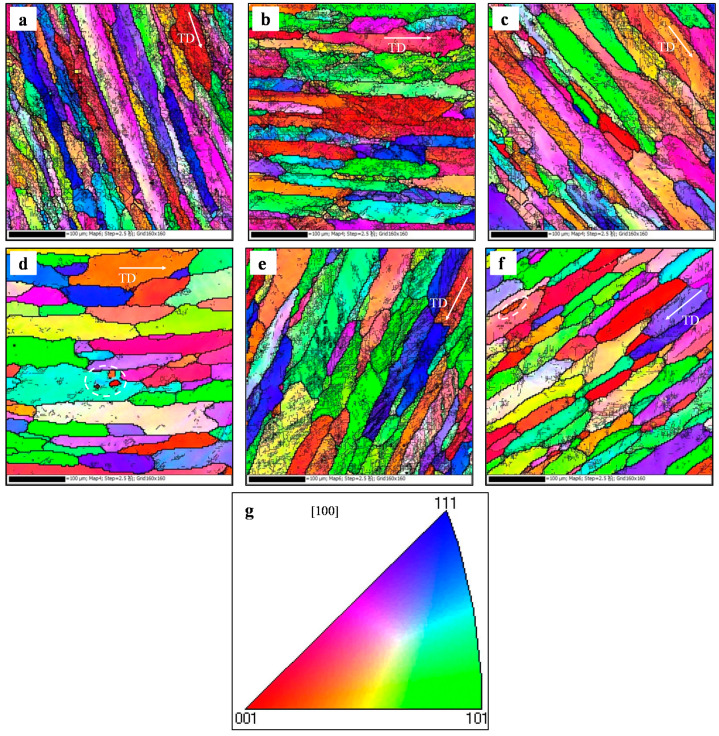
Inverse pole figure (IPF) maps of the stretched alloy at (**a**) *T* = 100 °C, $\dot{\varepsilon }$ = 0.0003 s^−1^; (**b**) *T* = 150 °C, $\dot{\varepsilon }$ = 0.0003 s^−1^; (**c**) *T* = 200 °C, $\dot{\varepsilon }$ = 0.0003 s^−1^; (**d**) *T* = 250 °C, $\dot{\varepsilon }$ = 0.0003 s^−1^; (**e**) *T* = 200 °C, $\dot{\varepsilon }$ = 0.003 s^−1^; and (**f**) *T* = 200 °C, $\dot{\varepsilon }$ = 0.00003 s^−1^. (**g**) Standard triangle for color code (Note: low-angle grain boundaries (LAGBs) and high-angle grain boundaries (HAGBs) are displayed by gray and black lines, respectively).

The inverse pole figure (IPF) maps of the stretched alloy at tested conditions are displayed in Figure 4. The grains are elongated along the tensile direction (TD) at different temperatures, as depicted in Figure 4a–d. The degree of grain elongation decreases with the raised temperature, i.e., the maximum grain elongation changes from 432 μm to 321 μm while the temperature is raised from 100 °C to 250 °C. Furthermore, substructures can be observed in Figure 4. The grain boundaries of substructures are low-angle grain boundaries (LAGBs). Here, the misorientation angles with 0° < *θ* < 15° and *θ* > 15° are referred to as LAGBs and high-angle grain boundaries (HAGBs), respectively. Meanwhile, the number of substructures decreases with the raised temperature. In addition, small fine grains around the elongated original coarse grains can be observed at 250 °C, which are marked with the white dotted line region in Figure 4d. The grain boundaries of these grains are HAGBs. Generally, LAGBs are formed by dynamic recovery (DRV), while HAGBs are formed by dynamic recrystallization (DRX) [36,37]. DRX grains are formed due to the rotation of small substructures [38,39]. This is consistent with those findings of other scholars [40,41]. In Figure 4e–f, the degree of grain elongation and the number of substructures decrease while the strain rate decreases from 0.003 s^−1^ to 0.00003 s^−1^. Additionally, some DRX grains can be found at 0.00003 s^−1^ in Figure 4f.

**Figure 5:**
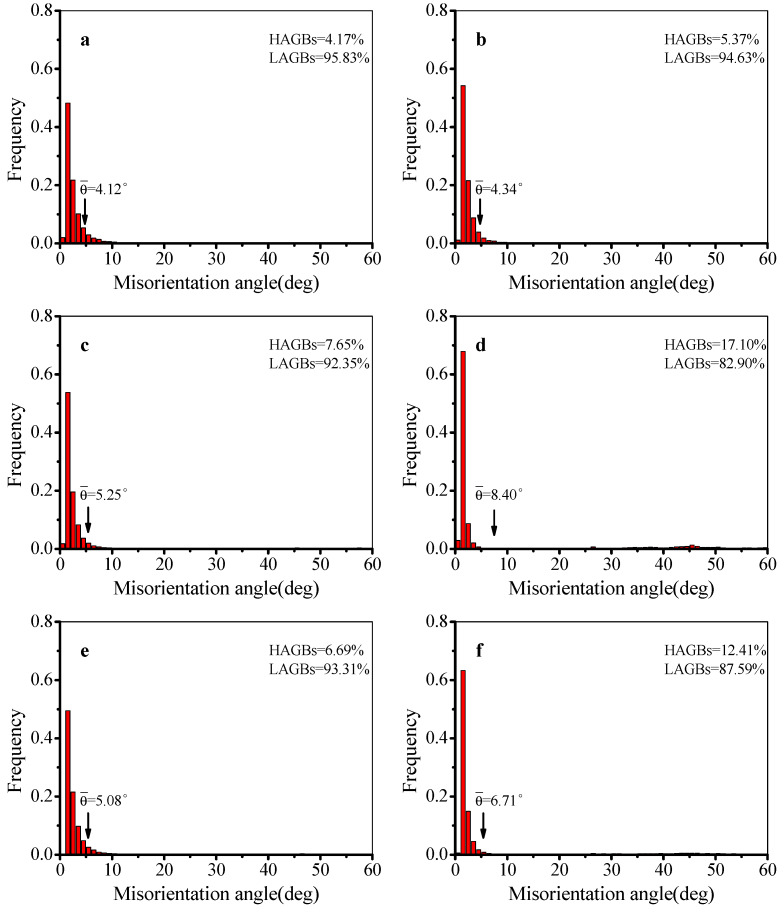
Distribution of grain boundary misorientation angles at (**a**) *T* = 100 °C, $\dot{\varepsilon }$ = 0.0003 s^−1^; (**b**) *T* = 150 °C, $\dot{\varepsilon }$ = 0.0003 s^−1^; (**c**) *T* = 200 °C, $\dot{\varepsilon }$ = 0.0003 s^−1^; (**d**) *T* = 250 °C, $\dot{\varepsilon }$ = 0.0003 s^−1^; (**e**) *T* = 200 °C, $\dot{\varepsilon }$ = 0.003 s^−1^; and (**f**) *T* = 200 °C, $\dot{\varepsilon }$ = 0.00003 s^−1^.

The distribution of grain boundary misorientation angles in different deformation conditions was evaluated by Channel 5 software (2009), as presented in Figure 5. In Figure 5a–d, the variations of mean misorientation angle and volume fraction of HAGBs increase with raising temperature at 0.0003 s^−1^. The maximum mean misorientation angle is 8.40° at 250 °C. The volume fractions of HAGBs are 4.17%, 5.37%, 7.65%, and 17.10%, respectively, at 100, 150, 200, and 250 °C. In Figure 5e,f, the variations of mean misorientation angle and volume fraction of HAGBs increase with raising strain rate at 200 °C. While the strain rate is reduced from 0.003 s^−1^ to 0.00003 s^−1^, the mean misorientation angle increases from 5.08° to 6.71°, and the volume fraction of HAGBs increases from 6.69% to 12.41%. 

According to Figure 5, the proportion of HAGBs is low in the tested conditions. This is because the stacking fault energy of Al–Cu alloy is high. The main softening mechanism during hot deformation is DRV. While the temperature is over 200 °C, the proportion of HAGBs increases, which reveals the increased DRX degree. Based on previous research [42], when the deformation temperature is more than half of the melting temperature of Al-Cu alloy, it is beneficial to the formation of DRX grains.

**Figure 6:**
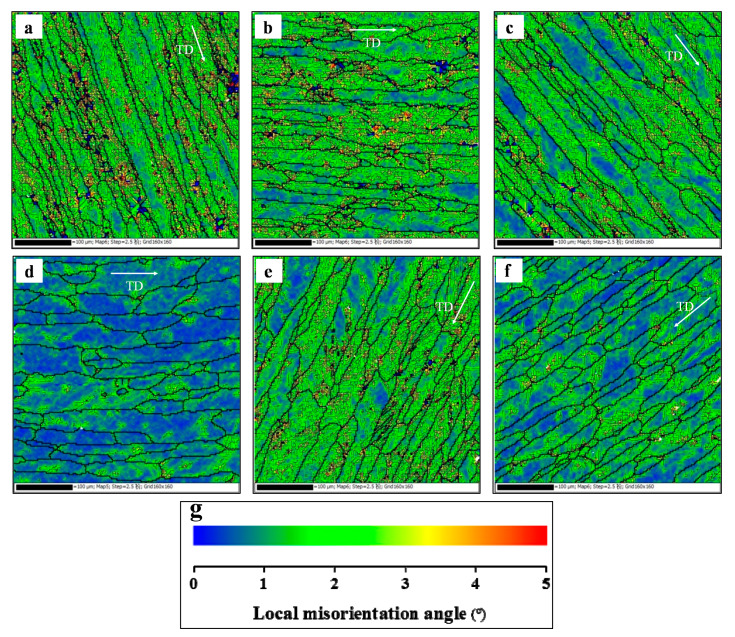
Kernel average misorientation (KAM) maps of the stretched alloy at (**a**) *T* = 100 °C, $\dot{\varepsilon }$ = 0.0003 s^−1^; (**b**) *T* = 150 °C, $\dot{\varepsilon }$ = 0.0003 s^−1^; (**c**) *T* = 200 °C, $\dot{\varepsilon }$ = 0.0003 s^−1^; (**d**) *T* = 250 °C, $\dot{\varepsilon }$ = 0.0003 s^−1^; (**e**) *T* = 200 °C, $\dot{\varepsilon }$ = 0.003 s^−1^; and (**f**) *T* = 200 °C, $\dot{\varepsilon }$ = 0.00003 s^−1^; (**g**) The scale label.

The kernel average misorientation (KAM) maps in the tested conditions are displayed in Figure 6. The values of KAM from 0° to 5° are marked as colors from blue to red. A large KAM value means a high geometrically necessary dislocation density [43,44]. In Figure 6a–c, it can be found that there are many green regions at different temperatures. Based on statistical analysis, the KAM values (mean local misorientation angle) are 2.00°, 1.77°, and 1.59°, respectively, at 100, 150, and 200 °C. When the temperature is raised to 250 °C, the dominant grains are blue color, and the value of KAM drops to 0.82°. The KAM maps of the stretched alloy at different strain rates are depicted in Figure 4e–f. As the strain rate decreases from 0.003 s^−1^ to 0.00003 s^−1^, the green regions decrease, and the value of KAM drops from 1.82° to 1.20°. The value of KAM is low at high temperatures or low strain rates, which reveals that the geometrically necessary dislocation density is low. This is because the DRV becomes more and more obvious, which accelerates the dislocation annihilation. Furthermore, the decreased substructures reduce the flow stress.

### 3.3. Precipitation Features

**Figure 7:**
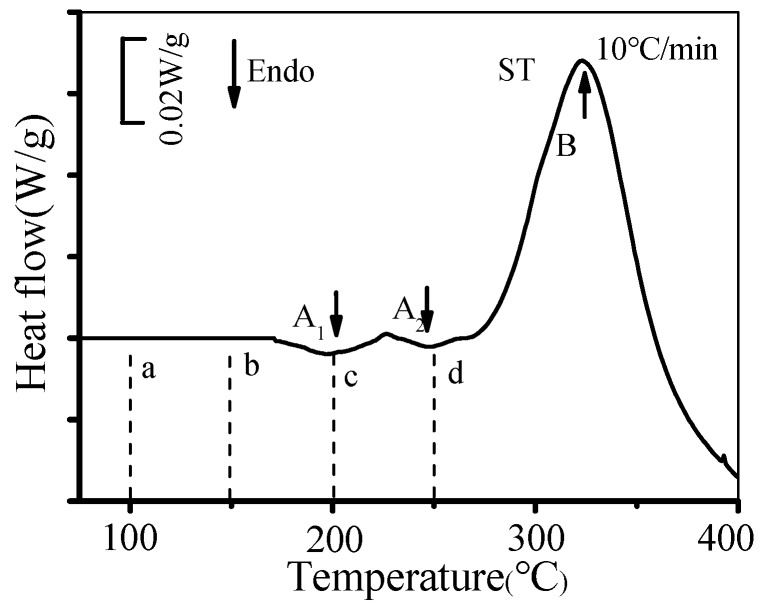
Differential scanning calorimetry (DSC) curves of the solution-treated alloy.

Figure 7 shows the DSC curves of the solution-treated alloy. Theoretically, four endothermic peaks and four exothermic peaks can be found in the DSC curves. Furthermore, four endothermic peaks represent the dissolution of Cu-rich clusters, G.P. zones, θ′′ and θ′ phases, while four exothermic peaks reveal the precipitation of G.P. zones, θ′′, θ′, and θ phases [45]. However, in actual experiments, not all the peaks can be fully found from the DSC curve due to the effect of heating rate. Rodríguez-Veiga et al. [46] found that few peaks can be observed when the heating rate is slow. In the present experimental curves, three peaks can be observed. Peak A_1_ resulted from the dissolution of G.P. zones, while peak A_2_ was induced by the dissolution of θ′′ phases. Peak B is related to the precipitation of θ′ phases. Because of the annihilation and overlap of peaks, the dissolution peaks of Cu-rich clusters, as well as the precipitation peaks of G.P. zones and θ′′ phases, cannot be easily observed. The temperature ranges of the dissolution peak of Cu-rich clusters, the precipitation peak of G.P. zones, and the precipitation peak of θ′′ phases are 50–85 °C, 105–125 °C, and 175–190 °C, respectively. The four tested deformation temperatures (100, 150, 200, and 250 °C) correspond to the four positions (a, b, c, and d) marked in the curves. Cu-rich clusters and G.P. zones formed after the alloy were heated to 100 °C and 150 °C, respectively. θ′′ phases are presented in the matrix when the alloy was heated to 200 °C and 250 °C. Thus, the influences of heating process before deformation on the type of precipitates are significant. This is further discussed following the TEM observation.

**Figure 8:**
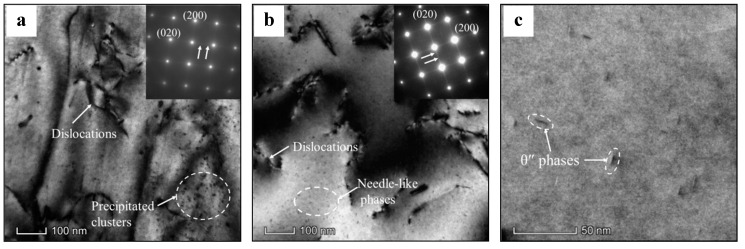
TEM images and selected area electron diffraction (SAED) patterns of the alloy heated to target temperatures of (**a**) 150 °C, and (**b**,**c**) 250 °C.

Figure 8 displays the TEM images and selected area electron diffraction (SAED) patterns of the heated alloy. Here, the heating rate is 10 °C/min, while the target temperatures are 150 °C and 250 °C. As depicted in Figure 8a, some dislocations can be found in the matrix at 150 °C. The Cu-rich clusters can be observed among dislocations. The SAED pattern reveals the continuous streaks along the {020}_Al_ position. These are some main characteristics of G.P. zones formed during heating [47,48]. When the target temperature is 250 °C (Figure 8b), dislocations can still be seen in matrix. In addition, some needle-like precipitates appear in the matrix. In the high-multiple TEM images (Figure 8c), the length of these phases is around 10 nm, and the type of these phases is θ′′, which is further confirmed by the SAED pattern. Discontinuous streaks appear along {020}_Al_ position, revealing the precipitation of θ′′ phases [49,50].

**Figure 9:**
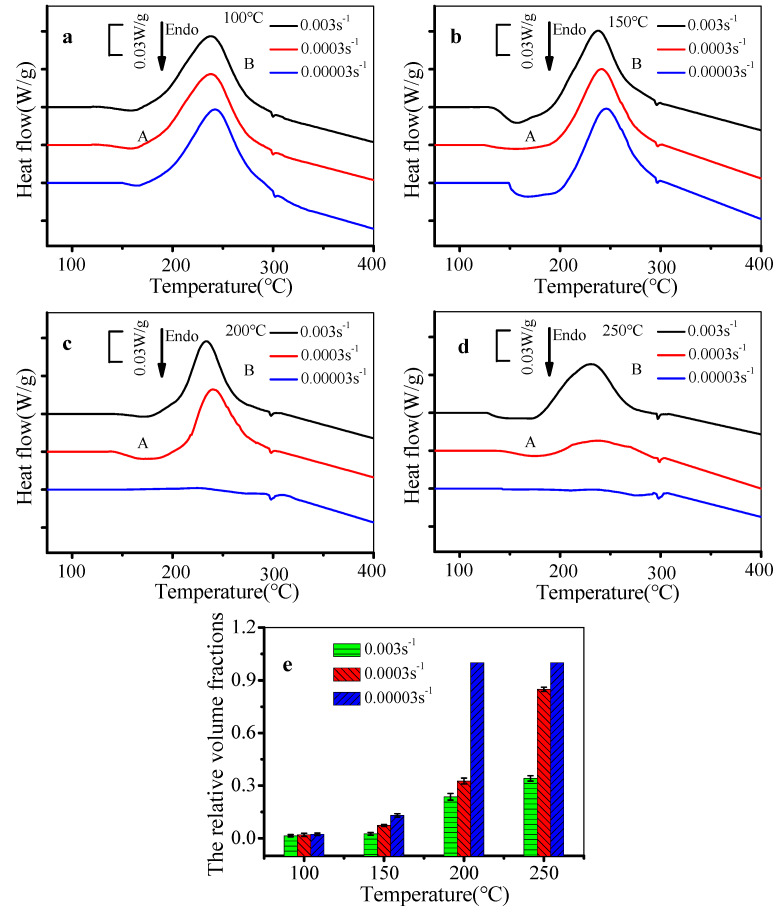
DSC curves and the relative volume fraction (RVF) of θ′ phases of the stretched alloy at (**a**) 100 °C, (**b**) 150 °C, (**c**) 200 °C, and (**d**) 250 °C. (**e**) The RVF of θ′ phases.

Figure 9 displays the DSC curves and the relative volume fractions (RVFs) of θ′ phases in the stretched alloy. Two characteristic peaks (endothermic peak A and exothermic peak B) appear in most DSC curves. The endothermic peak becomes large because of the overlap of peaks *A*_1_ and *A*_2_, which indicates the dissolution of G.P. zones and θ′′ phases. Peak B is correlated with the additional precipitation of θ′ particles. The decreased strain rate increases the characteristic temperature of peak A. This is because the migration of vacancies is more sufficient, and more G.P. zones transform to θ′′ phases. Thus, the stability of precipitates is increased when the strain rate is decreased. Therefore, peak A moves toward a high-temperature region, while the characteristic temperature of peak B moves toward a low-temperature region. This is because the nucleation of θ′ phases is affected by dislocations, and the deformation energy storage is low at low strain rates. The area of peak B decreases due to the decreased untransformed θ′′ phases. However, when the strain rate is 0.00003 s^−1^, it is hard to distinguish the endothermic peak and exothermic peak B from DSC curves at 200 °C and 250 °C (Figure 9c,d). This indicates that almost all the θ′′ phases transform to θ′ phases in the deformation process. 

The area of peak B in the tested deformation conditions was calculated. The RVF of θ′ phases can be evaluated as follows [51]:

(1) $$f=\frac{A_{1}-A_{2}}{A_{1}},$$

where *f* is the RVF of θ′ phases, A_2_ is the area of peak B in the tested deformation conditions, and *A*_1_ is the area of peak B in the solution-treated condition. The θ′ phases cannot be found in the solution-treated alloy, and the maximum content of θ′ phases could precipitate in the DSC experimental process. Thus, the area of peak B of the solution-treated alloy was taken as the denominator of Equation (1). The experimental results are shown in Figure 9e. At the same temperature, the RVF of θ′ phases of the stretched alloy increases with the decreased strain rate. The transformation of phases is more complete because of the longer deformation time at lower strain rates. At the same strain rate, the RVF of θ′ phases of the stretched alloy increases with the raised temperature. Firstly, the types of precipitates are affected by the heating process before deformation. θ′′ phases are present in matrix after heating to 200 °C and 250 °C, and they quickly transform to θ′ phases in the tensile deformation process. Secondly, the activation energy is high at high temperatures, which promotes the transformation of precipitates. 

**Figure 10:**
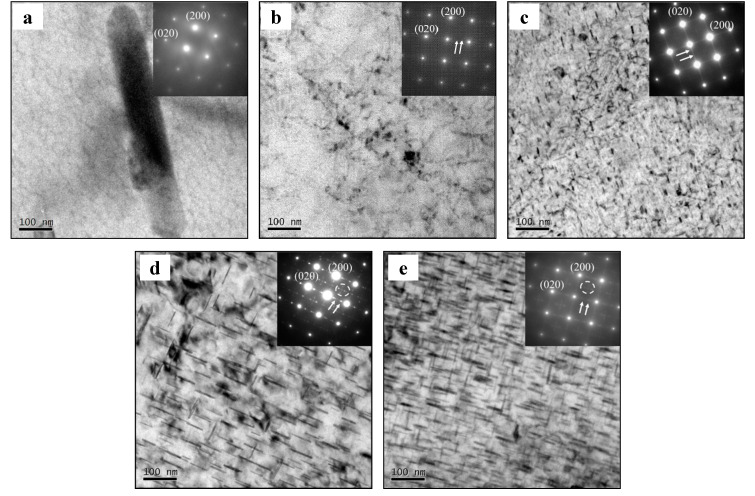
TEM images and SAED patterns of the stretched alloy at (**a**) *T* = 100 °C, $\dot{\varepsilon }$ = 0.0003 s^−1^; (**b**) *T* = 150 °C, $\dot{\varepsilon }$ = 0.0003 s^−1^; (**c**) *T* = 200 °C, $\dot{\varepsilon }$ = 0.0003 s^−1^; (**d**) *T* = 250 °C, $\dot{\varepsilon }$ = 0.0003 s^−1^; and (**e**) *T* = 200 °C, $\dot{\varepsilon }$ = 0.00003 s^−1^.

Figure 10 illustrates the TEM images and SAED patterns of the stretched alloy in tested deformation conditions. When the strain rate is 0.0003 s^−1^, the type, content, and size of precipitates are affected by the deformation temperature, as shown in Figure 10a–d. In Figure 10a, rod-like undissolved particles are present in the matrix at 100 °C. It is hard to distinguish the precipitates from the TEM image and SAED pattern. In Figure 10b, some clusters can be seen at 150 °C. Meanwhile, continuous streaks along the {020}_Al_ position in the SAED pattern reveal the appearance of G.P. zones. In Figure 10c, some needle-like precipitates appear at 200 °C, and their length range is between 10 nm and 18 nm. Furthermore, these precipitates are confirmed as θ′′ phases by the discontinuous streaks in the SAED pattern, which prefer to precipitate along the {020}_Al_ direction due to the effects of tensile force. This is the so-called stress orientation effect [44,52]. The content and size of needle-like precipitates increase while the temperature is raised to 250 °C (Figure 9d). The length of these precipitates varies from 41 nm to 92 nm. According to the SAED pattern, diffraction spots at the {110}_Al_ site indicate the formation of θ′ phases [44,46,53]. θ′ phases also prefer to precipitate along the {020}_Al_ direction. Except for the deformation temperature, Figure 10c,e shows that the type, content, and size of precipitates are also influenced by strain rate. The content and size of the needle-like precipitates rise as the strain rate is reduced from 0.0003 s^−1^ to 0.00003 s^−1^. The diffraction spots show that the precipitates are mainly θ′ phases. In addition, the distribution of these precipitates is not uniform, but most θ′ phases precipitate along the {020}_Al_ direction.

Therefore, the type, content, and size of precipitates are affected by strain rate and deformation temperature. According to the above analysis, in the heating process before tensile deformation, the transformation of precipitates (G.P. zones→θ′′ phases→θ′ phases) is more sufficient at higher temperatures. During tensile deformation, the content and size of precipitates further increase due to the enhanced activation energy at lower strain rates or higher deformation temperatures. Meanwhile, the transformation rate of precipitates is accelerated, resulting in the increased θ′ and θ′′ phases. However, θ′ and θ′′ phases prefer to precipitate along the {020}_Al_ direction due to the stress orientation effect, which results in the uneven distribution of phases.

### 3.4. Effects of Deformation Parameters on Mechanical Properties and Fracture Morphologies

**Figure 11:**
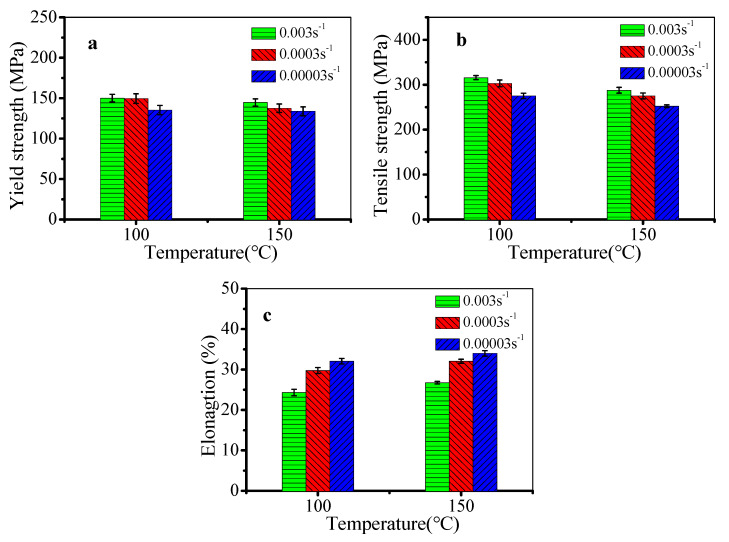
Mechanical properties of the stretched alloy at 100 °C and 150 °C: (**a**) yield strength; (**b**) tensile strength; (**c**) elongation.

Figure 11 shows the mechanical properties of the stretched alloy at 100 °C and 150 °C. The mechanical properties are usually affected by dislocation density, as well as the type, size, and content of precipitates. Based on the above analysis, the size and content of precipitates are relatively small at 100 °C and 150 °C, which slightly influence the mechanical properties. Thus, the mechanical properties are mainly affected by dislocation density. With the increased temperature or the decreased strain rate, the dislocation multiplication and work hardening weaken, leading to the decreased yield strength (Figure 11a). From Figure 11b, the tensile strength drops with the decreased strain rate or the increased temperature. This is because the DRV is more obvious at lower strain rates or higher temperatures. Then, the dislocation density is decreased. In Figure 11c, the elongation increases with the raised temperature or the decreased strain rate, because the plastic deformation is more uniform due to the enhanced DRV.

**Figure 12:**
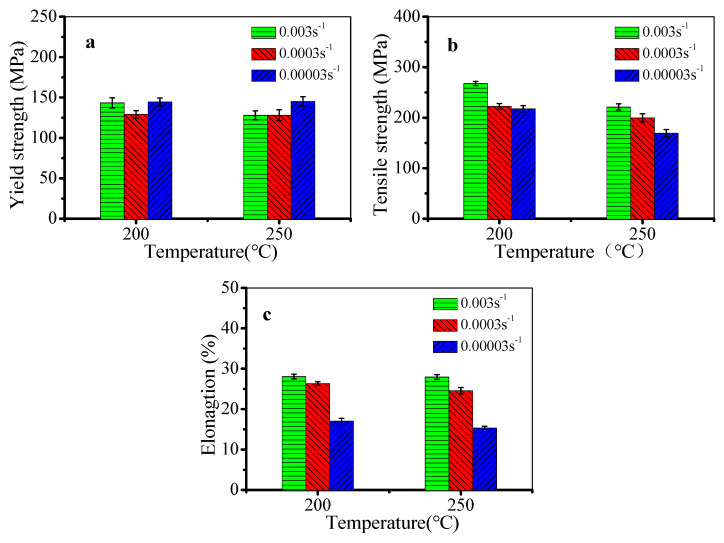
Mechanical properties of the stretched alloy at 200 °C and 250 °C: (**a**) yield strength; (**b**) tensile strength; (**c**) elongation.

Figure 12 shows the mechanical properties of the stretched alloy at 200 °C and 250 °C. Based on the above analysis, θ′′ phases precipitate in the heating process before deformation. During deformation, the transformation of precipitates is more sufficient, increasing the content of θ′ phases. Thus, the mechanical properties are mainly affected by the dislocation and precipitates. With the decreased strain rate, the dislocation multiplication and work hardening weaken. However, the contents of θ′′ and θ′ phases increase, which enhances the pinning effect of precipitates on dislocations. By combining the effect of strengthening from dislocation and precipitation, the yield strength firstly decreases and then increases (Figure 12a). In spite of the content and size of precipitates increasing with the decreased strain rate, the interaction between dislocations and precipitates is weak due to the decreased dislocation density. Thus, the tensile strength drops with the decreased strain rate or the increased temperature, as shown in Figure 12b. In Figure 12c, the elongation decreases with the decreased strain rate or the increased temperature. This is because the deformation time is long when the strain rate is low. The content and size of θ′′ and θ′ phases increase, and the incompatible deformation between phases and matrix increases. Therefore, the plasticity of the alloy decreases.

**Figure 13:**
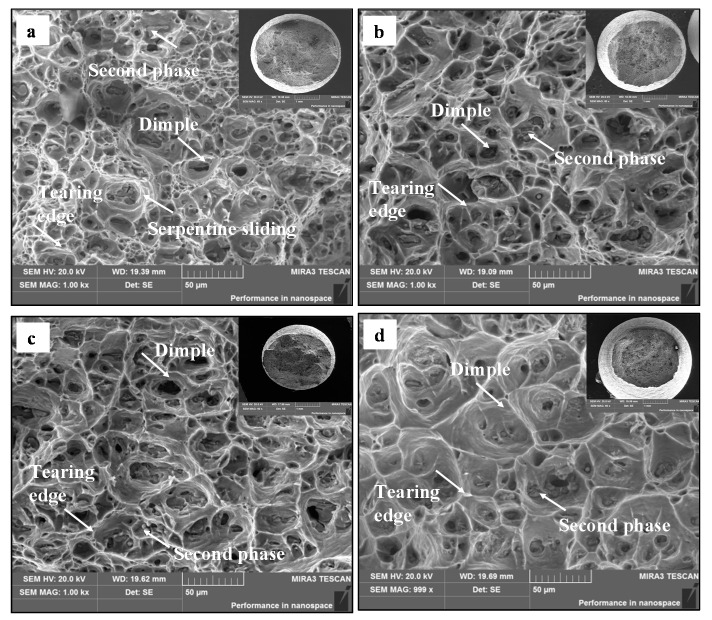
Fracture morphologies of the stretched alloy at (**a**) *T* = 200 °C, $\dot{\varepsilon }$ = 0.003 s^−1^; (**b**) *T* = 200 °C, $\dot{\varepsilon }$ = 0.0003 s^−1^; (**c**) *T* = 200 °C, $\dot{\varepsilon }$ = 0.00003 s^−1^; and (**d**) *T* = 250 °C, $\dot{\varepsilon }$ = 0.0003 s^−1^.

Figure 13 shows the fracture morphologies of samples. In Figure 13a, there are a lot of equiaxial dimples on the fracture surface, implying the shear fracture mode. The serpentine sliding and sharp tearing edges can be observed on dimple walls, i.e., the fracture mechanism is micro-void coalescence [54]. In addition, the second phase can also be seen at the bottom of the dimples. From Figure 13a–c, at 200 °C, the number and depth of dimples decrease with the decrease in stain rate. Meanwhile, the inhomogeneity of dimple sizes increases. This indicates that the plastic deformation is more uneven at a lower strain rate. Figure 13b,d shows the fracture morphologies at different temperatures when the strain rate is 0.0003 s^−1^. As the temperature is increased from 200 °C to 250 °C, the number of dimples decreases, and the inhomogeneity of dimple sizes increases. It can be concluded that the plastic deformation is more uneven with the increased temperature.

## 4. Conclusions 

The deformation behavior and precipitation features in a stretched 2219 aluminum alloy at intermediate temperatures were studied. Several important conclusions are given below.

- The flow stress monotonically rises at 100 °C and 150 °C due to the dominant work hardening. When the temperature is raised to 200 °C and 250 °C, the enhanced dynamic recovery reduces substructures. The dynamic recovery becomes more obvious at lower strain rates. Meanwhile, some dynamic recrystallization grains appear due to the rotation of small substructures. Thus, the true stress firstly increases and then keeps stable or drops with increasing true strain.
- The type of precipitates is greatly affected by the heating process before deformation. When the alloy is heated to 100 °C and 150 °C, Cu-rich clusters and G.P. zones appear. When the alloy is heated to 200 °C and 250 °C, θ′′ phases precipitate.
- The content and size of precipitates increase due to the enhanced activation energy at lower strain rates or higher deformation temperatures. Meanwhile, the transformation of precipitates is accelerated, and θ′ and θ′′ phases increase. However, θ′ and θ′′ phases prefer to precipitate along the {020}_Al_ direction due to the stress orientation effect, which results in the uneven distribution of phases.
- To alleviate work hardening and avoid the inhomogeneous precipitation of phases, the reasonable strain rate and temperature are about 0.0003 s^−1^ and 150 °C, respectively.
